# Community canteen services for the rural elderly: determining impacts on general mental health, nutritional status, satisfaction with life, and social capital

**DOI:** 10.1186/s12889-020-8305-9

**Published:** 2020-02-14

**Authors:** Xiaolei Wang, Minhui Liu, Yuchen Li, Chanyuan Guo, Chao Hsing Yeh

**Affiliations:** 1 Department of Nursing, School of Medicine, Hangzhou Normal University, Hangzhou, Zhejiang China; 2 Xiangya Nursing School, Central South University, No. 172 Tong Zi Po Road Yuelu District, Changsha, 410013 Hunan China; 3 Johns Hopkins University School of Nursing, Baltimore, MD USA

**Keywords:** Older adults, Nutrition, Health, Social capital, Satisfaction with life

## Abstract

**Background:**

The Chinese government is piloting canteen services for older adults, but few studies have explored the influence of canteen services on the health of these older adults. This study aimed to investigate the impact of canteen services on older adults’ general mental health, nutritional status, satisfaction with life, and social capital in rural areas.

**Methods:**

This study used a cross-sectional design. We selected 14 villages in Jinhua City, Zhejiang Province, China, including seven villages with canteen services and seven villages without canteen services. Participants were 284 senior older adults (aged 75~98), including 140 residing in villages with canteen services (Canteen Group [CG]) and 144 residing in villages without canteen services (Non-Canteen Group [NCG]). We also divided the CG into two sub-groups according to the funding sources (one receiving government support only, the other receiving government support plus enterprise donations). We used a self-designed questionnaire, including sociodemographics, diet-related items (e.g., satisfaction with the meals, diet expenditure, self-evaluation of meal nutrition), and the four scales including the Chinese version of the 12-item General Health Questionnaire (GHQ-12), the Chinese version of the Mini Nutritional Assessment Short-Form (MNA-SF), the Satisfaction with Life Scale (SWLS), and the Social Capital Questionnaire (SCQ).

**Results:**

The overall mental health, satisfaction with life, and social capital of the CG were better than the NCG (*P* <  0.05). The nutritional status of these two groups did not show a significant difference. Participants in the CG with financial support from local government and donations from an enterprise with a better dietary diversity exhibited a better nutritional status (*P* <  0.05); the average satisfaction with diet and self-evaluation of food nutrition of the CG were higher than the NCG (*P* < 0.05); the ratio of having a diet on time in the CG was higher than that of the NCG (*P* < 0.05).

**Conclusion:**

The free canteen services provided by the government can improve older adults’ satisfaction with life and diet, and mental health status and also enrich their social capital, but this still needs to be future evidenced. More financial support for canteen services is an essential component in promoting successful aging in China.

## Background

China is one of the fastest aging countries. In 2015, there were 144 million over 65 (one-fifth of the global older population) in China [1]. According to the Population Reference Bureau, by 2050 there will be more than 300 million people over 65 years of age in China [2]. The population over 80 years of age is expected to increase even more rapidly than any other age group [3].

To date, about 60% of the old population in China is scattered in rural areas [4]. Although most (70%) of the rural older adults have a pension, their median rural pension is just 60 Chinese Yuan Renminbi (CNY) per month (US$ 8.96). This pension is much less than the standard lowest life guaranteed amount—the minimum amount people should receive—in China (ranging from $CNY103 to 790/month [US$ 15.37 to 117.91], based on geographic locations) [5, 6]. So the pensions for rural older adults are usually not enough to afford basic living expenditures [5].

In the last three decades, as young rural Chinese migrated to big cities, “empty nests” emerged, comprising older adults with no children or whose children had already left home, including the older adults who lived alone (empty-nest singles), or with a spouse (empty nest couples) [7]. In 2013 the population of empty nests in China was over 100 million, and 45.6% of these were rural [8]. In the empty nest families, traditional family roles have been weakened because of the absence of young family members who can care for older adults [9]. This lack of support has a particularly negative effect on the older adults (e.g., loneliness, poor satisfaction with life, and low living standards) [10], especially for senior older adults (those 75 years of age and above), with decreased activities of daily living (ADL) and the need for increased assistance [11]. According to a national survey in China in 2005, one third of senior older adults in rural areas need daily care, including food support services [12]. Cooking independently can be a burdensome activity for older adults, particularly for those living alone [13].

In many other countries, many services are available to provide meals for older adults who need nutritional support to ensure that older adults have adequate access to nutrition [14]. Meals-on-Wheels, the most widely recognized home delivery meals (HDM) service, is available in many countries. It can either provide individuals meals at home or by congregate meals (meals are provided in group settings such as senior citizen centers, supporting people with the opportunity to socialize when eating together). HDM can improve dietary intake [13] and decrease the institutionalization of older adults; it is also a potential mechanism for decreasing healthcare expenditures [15].

To address this situation in China, scholars and policymakers are seeking optional solutions for further improvement of the quality of care for older adults. In 2015, the national government of China, through the Ministry of Civil Affairs and the China Development Bank, launched a policy named “Financial Support for the Construction of Social Service System for the Older Adults.” [16] A key part of the new system was the establishment of community canteens for older adults. The canteen services were to be organized and financially supported by the local government. In compliance with this policy, many local governments were piloting community canteens for their older population [17].

Although home-delivered meals appear to be an appropriate solution for older adults who are often frail and nutritionally at risk, there is only scattered and limited evidence documenting their efficacy and effectiveness. A review showed that two studies found that the HDM programs significantly improved diet quality, increased nutrient intake, reduced food insecurity and nutritional risk among participants, and also brought other beneficial outcomes, such as increased socialization opportunities, improved dietary adherence, and higher quality of life [18]. To the best of our knowledge, there are no reports published about the impact of community canteens on senior older adults’ (those 75 years of age and above) health in China. In this study, we aimed to evaluate the effect of the new rural community canteens on the senior older adults in China in the following aspects: general mental health, nutritional status, satisfaction with life, the social capital, and diet-related factors (satisfaction with the meals, diet expenditures, self-evaluation of meal nutrition, regularity of eating and service evaluation). We hypothesized that the recipients of canteen service would show significantly better health outcomes than the non-recipients.

## Methods

### Study design and settings

To determine the impact of community canteen services on the older adults, a cross-sectional observational study was conducted between July and October 2017 in villages of Jinhua, located in the center of Zhejiang Province near the southeast coast of China. Zhengjiang is one of the fastest-growing provinces in China, with a gross domestic product (GDP) of 5180 billion CNY (773.13 billion US dollars) in 2017. Jinhua City is ranked as moderate in economic development. The population of Zhejiang Province was about 57 million in 2017, including 11 million older people (≥ 60 years of age), about 1.7 million of whom were over 80 years old. As a developed province, Zhejiang has piloted the community canteen services for several years and had established 14,208 canteens by the end of 2017 [17].

### Community canteen services

The community canteen services are supported financially by the local government. In Zhejiang, the Civil Affairs Bureau has cooperated with the local government at the village or community level in the establishment and operation of the canteens by the provision of grants for building and operating costs, including paying the chefs, and the cost of the meals. The canteen services such as hygiene and meals are evaluated periodically by the Civil Affairs Bureau, with performance determining subsequent financial bonuses or penalties given.

The local chefs decide which food to offer, with a variety of food being available if an individual canteen receives additional funding donated from an enterprise such as a firm. The canteens offer the opportunity for rural older adults to eat lunch and dinner together each day. The eligible age for participation in the community canteen services is determined by the local government at the village level. Typically, the age requirement is 75 years of age in a relatively prosperous village, and 80 years of age in a poorer village, and usually all the older adults who met the age requirements would receive the canteen services. Depending on the circumstances of each canteen, an older adult can eat at the canteen for free (if the village supplements the payments made by the Civil Affairs Bureau), or for a nominal contribution to costs.

### Participants

We selected seven villages that had community canteen services (Canteen Group [CG]) and then matched these villages with the seven closest villages that did not offer the services (Non-Canteen Group [NCG]). In each village, we recruited all the target older residents by convenience and snowball sampling strategies with assistance from the leaders of the villages. Older adult residents were eligible for the study if they met the following criteria: (1) 75 years or older, (2) community-dwelling participants who lived in the selected area for more than 5 years, (3) were able to communicate easily, and (4) were willing to participate. Residents were excluded if they: (1) had severe physical illness (e.g., severely disabled and could not walk) or mental illness; (2) were unwilling to participate; or (3) could not communicate, even with the assistance of the investigator (such as being illiterate with severe hearing loss, or illiterate with obvious language difficulties). A total of 300 residents were approached and given the questionnaire; 284 responded comprehensively (see data analysis below) and were, therefore, survey participants, giving a final response rate for the survey of 94.7%.

### Data collection

The older adults who met the inclusion criteria were informed about the study details and were asked to give their consent to participate in the survey. Participation was voluntary, and the questionnaire was anonymized. The questionnaire was distributed by investigators at local communities (for participants with vision problems who could not read the questionnaire, the investigator read items and recorded the participants’ responses on the questionnaire). Data collection took approximately 15 min for each participant. Once data collection was completed, the questionnaires were double-checked to ensure completion, integrity, and accuracy of the information. Participants were given a 10 CNY (US$ 1.49) gift card as compensation for their time.

### Measurements

A structured questionnaire comprising 45 items was piloted with 40 older adults and then modified based on their feedback before collecting the data. The questionnaire inquired about participants’ sociodemographics and other information, general mental health status, nutrition status, and satisfaction with life and social capital.

#### Sociodemographics and other information (10 items)

We developed 10 items to collect sociodemographic and other diet-related information based on the recommendations from the literature [19]. Sociodemographic characteristics were age, gender, marital status (married or not married, which included never married, losing a partner, divorce), education (illiterate, middle school, senior middle school, undergraduate or higher), income (per month < 300 CNY, 301~1000 CNY, 1001~2000 CNY, 2001~3000 CNY, > 3000 CNY). Diet-related information included the following: diet expenditures (“How much is your average daily diet expenditure including lunch and dinner?”), satisfaction with the meals (“How satisfied are you with the meals?”) on a scale from 1 (completely unsatisfied) to 5 (completely satisfied), self-evaluation of meal nutrition (“How do you evaluate the nutrition of meals?”) on a scale from 1 (very poor) to 5 (very good), the regularity of eating (“Is your meal time regular?” with responses of irregular and regular), and an evaluation of the canteen services (“What do you think are the most obvious benefits you get from the canteen services?” This is a multiple-choice question, with choices such as more regular diet time, better nutrition, fresher diet, more convenience, etc.).

#### General mental health status (12 items)

To evaluate the general mental health status of the participants in the prior month before the study, we used the Chinese version of the 12-item General Health Questionnaire (GHQ-12) [20], which was adapted from the general health questionnaire developed by David Goldberg in 1972 [21]. The total scores ranged from 0 to 12, with higher scores indicating worse mental health [20]. The cutoff of 3 had a sensitivity of 76.85% and a specificity of 77.73%. Participants with a total score of 4 or higher were classified as having poor mental health status and participants with a total score of 3 or lower were classified as having good mental health [20].

#### Nutritional status (6 items)

We used the Revised Mini Nutritional Assessment Short-Form (MNA-SF), a practical tool for identifying nutritional status among older adults [22]. The first five items of the revised MNA-SF are unchanged from the original MNA-SF; the sixth question in the revised MNA-SF addressed either body mass index or calf circumference, depending on the feasibility of taking the measurements. In this study, we used the calf circumference as the sixth question. The revised MNA-SF had three classifications: 0–7 points indicate being malnourished, 8–11 points indicated being at risk of malnourishment, and 12–14 points indicated being well-nourished. This Chinese version of the revised MNA-SF has been tested as highly reliable and valid [23].

#### Satisfaction with life (5 items)

The Satisfaction with Life Scale (SWLS) was developed by Diener et al. [24]. It primarily asks five direct questions about satisfaction with life (e.g., “In most ways my life is close to my ideal,” “the conditions of my life are excellent”), which are answered on a 7-point Likert scale, from 1 “strongly disagree” to 7 “strongly agree.” The overall score ranges from 5 to 35, with higher scores indicating a higher level of satisfaction with life. We used the Chinese version of the SWLS, which has been tested and found to be highly reliable and valid [25].

#### Social capital (12 items)

Social capital is an important positive impact factor for health [26]. The Social Capital Questionnaire (SCQ) developed by Yang has acceptable reliability and validity [27] and was used to evaluate each participant’s social capital status. There are three domains with a total of 12 items. The aspects pertained to cognitive social capital based on binary responses (yes/no) to four questions (e.g., “In general, would you believe most people?”), three questions on social participation (e.g., “How many times did you participate in leisure or entertainment activities organized by your community during the past year?”), and five questions on social network (the number of good friends, colleagues before, helpful neighbors, close relatives and cooperative partners). A higher total score meant better social capital.

### Data analysis

We performed statistical analyses using SPSS (Version 22.0 for Windows, IBM, New York, NY, USA) statistical software. From the initial 300 questionnaires, eight were excluded for low-quality data (e.g., all answers were the first choice of the question, answers had a pattern such as 1, 2, 1, 2, ….). Eight were excluded for missing data. Thus, there was a final sample size of 284. For participants with 1 item missing in SCQ (2 [0.7%]) and GHQ-12 (1 [0.3%]), we completed their questionnaires with the average item score.

Descriptive statistics were presented as the mean and SD for continuous variables, and the number (percentage) for categorical variables. Means for age, satisfaction with life, nutritional assessment, social capital, general health, satisfaction with the meals, and self-evaluation of meal nutrition were compared between the CG and NCG using independent *t*-tests. The categorical data of gender, marital status, and regularity of eating were compared between the two groups using the chi-square test. The categorical data of education and income were compared by the Mann-Whitney test.

To compare nutritional differences among three groups (the CG with government support and enterprise donation, the CG with government support only, and the NCG), we used ANOVA and Fisher’s Least Significant Difference (LSD) tests. Statistical significance was set at *P* < 0.05.

## Results

### Sociodemographic characteristics

A total of 284 valid questionnaires were obtained, representing 140 older adults residing in the villages with canteen services (CC) and 144 older adults residing in the villages without canteen services (NCC). Among these 284 older individuals, the average age was 83.07 ± 4.19 years, with a range of 75 to 98 years; 136 (47.9%) were male, and 148 (52.1%) were female; 130 (45.8%) were non-single (married), and 154 (54.2%) were single (including unmarried, divorced and widows/widowers) (Table 1). Of the 284 older adults, 187 (65.8%) were illiterate; only 1 participant (0.4%) had a higher education (undergraduate); 205 (72.2%) of the older adults had an income lower than 1000 CNY/month (US$149.25) (Table 1).

**Table 1:** Comparison of sociodemographic characteristics of Canteen and Non-Canteen groups

Sociodemographics	Total	Canteen Group	Non-Canteen Group	*P* values
Age (mean, SD)	83.07 (4.19)	83.54 (4.55)	82.60 (3.77)	> 0.05
Gender (n, %)				> 0.05
Male	136 (47.9)	63 (45.0)	73 (50.7)	
Female	148 (52.1)	77 (55.00)	71 (49.3)	
Marital status (n, %)				> 0.05
Non-single ^a^	130 (45.8)	59 (42.1)	71 (49.3)	
Single	154 (54.2)	81 (57.9)	73 (50.7)	
Education (n, %)				> 0.05
Illiterate	187 (65.8)	100 (71.4)	87 (60.4)	
Primary school	76 (26.8)	30 (21.4)	46 (31.9)	
Middle school	19 (6.7)	8 (5.7)	11 (7.6)	
Senior middle school	1 (0.4)	1 (0.7)	0 (0.0)	
Undergraduate or higher	1 (0.4)	1 (0.7)	0 (0.0)	
Income/month (CNY) (n, %)				> 0.05
< 300	94 (33.1)	50 (35.7)	44 (36.6)	
300–999	111 (39.1)	50 (35.7)	61 (42.4)	
1000–1999	58 (20.4)	32 (22.9)	26 (18.1)	
2000–2999	11 (3.9)	4 (2.9)	7 (4.9)	
≥ 3000	10 (3.5)	4 (2.9)	6 (4.2)	

Notes: *SD* standard deviation; ^a^Non-single includes living as part of a couple; single includes the divorced, never married, or widowed

There were no significant differences in age, gender, marital status, educational level, or income between the CG and NCG groups (Table 1).

### Comparisons of health status and social capital

The health status (general mental health status, nutritional status, satisfaction with life) and social capital of the CG and NCG groups are presented and compared in Table 2. The older adults with canteen services showed better mental health, greater satisfaction with life, and richer social capital than the older adults without canteen services (Table 2).

**Table 2:** Comparison of four indices (general mental health, nutritional status, satisfaction with life and social capital) of health status in Canteen and Non-Canteen groups

Variables	Canteen Group (*n* = 140) Mean (SD)	Non-Canteen Group (*n* = 144) Mean (SD)	*P* values
General mental health (GHQ-12)	1.39 (1.95)	1.93 (2.36)	0.038
Nutritional status (MNA-SF)	12.46 (2.03)	12.31 (2.22)	> 0.05
Satisfaction with life (SWLS)	26.80 (4.68)	22.88 (5.85)	< 0.001
Social capital (SCQ)	17.89 (1.38)	17.48 (1.64)	0.026

Notes: *SD* Standard Deviation; *GHQ-12* the 12-item General Health Questionnaire; *MNA-SF* the Revised Mini Nutritional Assessment Short-Form; *SWLS* Satisfaction with Life Scale; *SCQ* Social Capital Questionnaire

In contrast, nutritional status was not significantly different between the CG and NCG groups (Table 2). The detailed MNA-SF scores were as follows: (i) in the CG group, 4 older adults experienced malnutrition (< 8), 42 were at risk of malnutrition (8–11), and 94 were in good nutritional status (12–14); (ii) in the NCG group, the corresponding numbers of older adults were 7, 45, and 92. There was no significant difference in the ratio of malnourished individuals between the two groups (*χ*^*2*^ = 0.887, *P* = 0.642).

### Comparison of nutritional status in three groups defined by funding sources and daily meal costs

Due to the insignificant difference in nutritional status between the CG and NCG groups when all community canteen services were considered together, we then examined the nutritional status in more detail. We investigated whether nutritional status differed based on the amount of funding for the canteen services by dividing the CG into two sub-groups: (1) CG with financial support from local government and donation from an enterprise (daily meal costs per person 10~15 CNY (US$1.49~2.24); (2) CG with only financial support from local government (daily meal costs per person 5 CNY (US$0.75)); and for the NCG, they paid for their meals by themselves. The daily meal costs per person were as follows: 8.3% (12/144) < 3 CNY (US$0.45), 48.6% (70/144) 3~5 CNY (US$0.45~0.75), 25% (36/144) 6~10 CNY (US$0.90~1.49), 16% (23/144) 11–20 CNY (US$1.64~2.99), and 2.1% (3/144) > 20 CNY [US$2.99]).

As indicated by the MNA-SF scores, the nutritional status was significantly different between these three groups (Table 3). Further analysis by LSD multiple comparisons showed that the older adults in sub-group 1 (CG with government and enterprise support) had a significantly better nutritional status than the older adults in sub-group 2 (CG with government support only) and the older adults in NCG (Table 3). The nutritional status of the older adults in sub-groups 2 and NCG was not significantly different (Table 3).

**Table 3:** Comparison of nutritional status among three groups defined by funding sources and daily meal costs

Groups	n	Nutritional status	LSD multiple comparisons between groups
1 Canteen Group Government support plus enterprise donation	40	13.28 (1.32)	1 v 2*
2 Canteen Group Government support only	100	12.13 (2.17)	1 v 3*
3 Non-Canteen Group	144	12.31 (2.22)	2 v 3

Notes: *LSD* Least Significant Difference; Data for nutritional status are means and standard deviation;

*: *P* < 0.05

### Comparisons of other diet-related items in two groups

The older adults in the community canteen group reported significantly higher satisfaction with meals, self-evaluation of the meal nutrition, and regularity of meals than the older adults without a community canteen (*p* < 0.001 for all three aspects) (Table 4).

**Table 4:** Comparison of satisfaction with the meals, self-evaluation of the nutrition of meals and regularity of eating between Canteen and Non-Canteen groups

Diet-related items	Canteen Group (*n* = 140)	Non-Canteen Group (*n* = 144)	*P* values
Evaluation (mean, SD)			
Satisfaction with the meals	4.72 (0.51)	3.66 (0.85)	< 0.001
Self-evaluation of the nutrition	3.42 (0.72)	2.50 (1.07)	< 0.001
Regularity of eating (n, %)			
Regular	136 (97.1%)	115 (79.9%)	< 0.001
Irregular	4 (2.9%)	29 (20.1%)	

Notes: *SD* Standard Deviation

### Most cited benefits of community canteen services

When asked about the benefits of the community canteens, 122 participants (87.1%) cited increased convenience and 118 (84.2%) cited the fresher diet; 114 (81.4%) participants cited better nutrition status of diet; 98 participants (70.0%) cited improved regularity of eating, compared to not eating in the canteen (Tables not shown).

## Discussion

This study explored the influence of community canteen services for rural older adults in China. We found that satisfaction with life, general mental health, diet-related factors, and social capital of the CG were better than the NCG.

The older adults in CG were more satisfied with life than were those in the NCG. A study conducted in Ecuador showed that the higher levels of satisfaction with life in Ecuadorian seniors were associated with healthier eating habits (e.g., no lunch skipper, more nutritional diet) [28], so changes in the eating habits resulting from the canteen services might be one of the reasons for higher satisfaction with life in CG. We also considered the welfare aspect of being an important reason, because the free (or greatly subsidized) food supplied by the canteen services could save the older adults a significant portion of their usual living expenses and reduce their meal preparation burden, so the canteen services might make the older adults feel more positive about life.

Malnutrition is especially harmful to older adults [29]. It may cause many non-specific symptoms such as poor appetite, chronic fatigue, and a feeling of ill health; it is also a major risk factor for illness, poor recovery, and increased cost of health resources [29]. Three recent studies reported the prevalence of malnutrition (1.3 to 3.2%) and risk of malnutrition (19.3 to 24.4%) in China [30–32]. An 11-year population-based longitudinal study in Sweden also showed the prevalence of malnutrition (1.7%) and risk of malnutrition (24.7%) [33]. Our study found that the prevalence of malnutrition (3.9%) and the risk of malnutrition (30.6%) was higher than those studies. We suggest that two main reasons explain the difference: age and location. In the four studies mentioned above, the participants were mostly over 60 years old; however, in our study, participants ranged in age from 75~98 years, with an average age of 83.07 ± 4.19 years. Aging is associated with distinct changes in gastrointestinal function and body composition, which might disrupt digestion or absorption [34]. When considering location, our study was conducted in rural areas, which have been shown to be a risk factor for malnutrition [35].

A systematic literature review showed that the home-delivered meal service in other countries was able to promote beneficial results in nutritional intake in community-living older adults [36]. In our study, however, the nutritional status as measured by the Revised MNA-SF between the CG and the NCG was not significantly different. To explain this unexpected result, we further examined the dietary expense. In most of the CG (CG with government support only), the average diet expenditure was about 5 CNY (US$ 0.75) per person per day. In the NCG, 43.1% of the older adults spent more than 5 CNY per day on their diet, and 56.9% of them spent less than 5 CNY. We speculated that the diet expenditures of CG and the nutritional status might have no particular advantages over that of the NCG. A more detailed comparison showed that there was indeed a significant difference in nutritional status between groups: the nutritional status of the CG with additional donations from an enterprise was significantly better than that of the CG without donation and the NCG.

The diet expenditures of 5 CNY per person per day could limit dietary diversity. For example, in 2017, the average price of pork was 28.58 CNY per kilogram [37], so the older adults in CG might still not intake enough nutrients. If a canteen could get extra donations from an enterprise and more money could be put into the canteen services, the nutritional status of older adults might be better as they could afford a variety of food. Overall, when all community canteens were considered together, the older adults felt that meals were more convenient, the food was fresher than that cooked by themselves, and the canteen services allowed them to eat more regularly than before. Regular eating habits can reduce the incidence of mental illness [38]. Proper and balanced nutrition for older adults is essential not only to maintain a healthy life but also to improve the quality of mental health [39]. Thus, canteen services might help them build healthier eating habits and bring about a positive feeling.

Social capital—a way of conceptualizing and measuring the social environment—has different definitions depending on the researchers’ perspectives [40, 41]. In this study, we adopted the definition of “features of social organization, such as trust, norms, and networks that could improve the efficiency of society by facilitating coordinated actions” [41]. It included two major dimensions: the structural social capital (the number of social interactions: externally observable relationships among people) and the cognitive social capital (quality of social interactions: norms, beliefs, and values that determine mutual benefit) [42]. A systematic review of 56 studies found that social capital has a positive effect on health [26]. Zhu et al. concluded that home-delivered meal programs could increase socialization opportunities [18]. In our study, we also found that the social capital of the community canteen group was significantly stronger than that of the no-canteen group. This difference might be explained by the fact that with the canteen services, the older adults had more opportunities to communicate with others and make friends with each other, thus increasing their social interactions and decreasing their negative emotions such as loneliness.

In this study, the general mental health of the older adults in CG was significantly higher than that in NCG. We thought this outcome could be explained in several aspects. First, when participants had a high status of satisfaction with life, social capital was correlated with better mental health [26, 43]. Second, eating alone is associated with mental problems, such as depression [44]. The canteen services provided a chance to eat together, which might be beneficial for older adults’ mental health. Third, as mentioned above, regular eating habits can reduce the incidence of mental illness [38], so a regular eating status in CG might be related to better mental health in this study.

To the best of our knowledge, this is the first study undertaken to investigate the impact of community canteen services on general mental health, nutritional status, satisfaction with life and social capital among rural older adults in China. Our results may serve as preliminary for future studies using more robust methods to investigate the relationships among canteen services and health in geriatric populations.

This study also has several limitations. First, it was a cross-sectional study and only evaluated relationships at specific time points rather than over an extended period. Whether the canteen services could improve the health of the recipients needs to be evidenced by future studies, especially with longitudinal research designs. Second, the canteen service policies may be different for different provinces, but this study focused on senior older adults residing in villages near Jinhua City in Zhejiang Province, which is one of the top four provinces in terms of gross domestic product in China. Thus, the results may not be broadly applicable to other provinces, especially to those in poorer regions of the country. To resolve this limitation, future studies should be conducted in other provinces, considering the local policy for older adults and the local economic status. Third, we were not able to measure aspects such as the protein and other nutrients older adults’ daily intake and other potential confounding factors that could affect satisfaction with life, mental health, and nutritional status, so the results may be biased. Forth, we only measured the benefits of the canteen services, not what people did not like. Future studies should examine these aspects more deeply. Finally, we note that data were self-reported; thus, the older adults in the study may have made socially desirable responses.

## Conclusions

Based on this survey, the older adults in the CG exhibited a status of better mental health, greater satisfaction with life, and richer social capital; they also showed greater satisfaction with meals, higher evaluation of the meal nutrition, and more regular diet habits. Although the nutritional status of the CG and NCG did not show a significant difference, the sub-group in CG with the extra donation and higher diet expenditures exhibited a better nutritional status. The canteen services can improve older adults’ general mental health, satisfaction with life and social capital, but this needs to be future evidenced by more studies. Additionally, The canteen services with more financial support are essential components in promoting successful aging in China.

## Data Availability

The dataset used and analyzed during the current study is available from the corresponding author upon reasonable request.

## Abbreviations

ADL: Activities of daily living
CG: Community canteen group (Canteen Group)
CNY: Chinese Yuan Renminbi
GHQ: General Health Questionnaire
HDM: Home-delivered meals
LSR: Life Satisfaction Rating Scales
MNA-SF: Mini Nutritional Assessment Short-Form
NCC: Villages without canteen service
NCG: No community canteen group (Non-Canteen Group)
SCQ: Social Capital Questionnaire
SWLS: Satisfaction with Life Scale
